# Heterogeneous Ice Nucleation by Graphene Nanoparticles

**DOI:** 10.1038/s41598-020-66714-2

**Published:** 2020-06-16

**Authors:** Mohammad Joghataei, Fatemeh Ostovari, Samira Atabakhsh, Nafiseh Tobeiha

**Affiliations:** Department of Physics, Yazd University, Yazd, Iran

**Keywords:** Atmospheric chemistry, Imaging techniques, Graphene

## Abstract

Nanostructure, chemical composition and size distribution of aerosols have prime important effects on their efficiency in heterogeneous ice nucleation (HIN). The ice nucleation usually requires active sites in the aerosols in order to act as ice nuclei (IN). In this study, HIN and probable active sites of the graphene-graphene oxide nanoparticles (GGON), obtained from graphite oxide by low temperature thermal shock (LTTS), were investigated. Characteristics and size distribution of the GGON were identified using scanning electron microscope (SEM) and image processing of the results, Fourier transform infrared spectroscopy (FTIR), Raman spectra and X-ray diffraction (XRD) of their sheets. The FTIR spectra indicate stronger carbon-oxygen bonds in the samples obtained by LTTS. In addition, maximum size distribution of the GGON was ranged around 160–180 nm. After introducing these particles in the cloud chamber, HIN has occurred and ice crystals were formed. Size distribution of crystals were obtained from image processing of the plates, where covered by a thin layer of Formvar, showed the number of ice crystals in the GGON were increased as temperature increased from −20 °C to −10 °C. In addition, two possible mechanisms of asymmetry and deformation in ice crystals of the GGON were described.

## Introduction

Cloud-aerosol interactions in the Earth’s atmosphere have several important effects on weather and climate system. First of all, these interactions can change size distribution of aerosols, whose impact on both incoming shortwave and outgoing longwave radiations are evidenced^1–3^. Secondly, more than 50% of Earth’s precipitation originates in the ice phase^4^ and particles that can serve as ice nuclei (IN) are essential in microphysics of cloud and precipitation. In addition, nowadays humans try to modify clouds in order to increase his water resources, and in this context artificial aerosols that contribute in cloud microphysics are desired in cloud seeding. So the aerosol-cloud interactions and consequently their effects on weather, climate and climate change are among important environmental global issues^3–5^.

Aerosol particles (AP) are injected into the atmosphere from natural and anthropogenic sources via different mechanisms, and they can experience different kinds of aging and phase change processes during various atmospheric conditions. The process of transformation of AP to droplet is primarily dependent on their size, shape and chemical composition in the process known as activation of AP. Kohler theory describes the competing effects involved in cloud droplet activation^6,7^. Aerosol particles with especial characteristics, which can act as cloud condensation nuclei (CCN) at the low supersaturations (SS) typical for atmospheric clouds, can be activated. On the other hand, heterogeneous ice nucleation (HIN) requires usually an insoluble AP to serve as an IN that initiates the ice phase through direct deposition of water vapour, freezing through aqueous medium and via contact, immersion or condensation of specific AP^7–10^. In deposition mode, ice crystals (IC) become larger by deposition of the water molecules onto the surface of the IN at the expense of the nearby liquid droplets in the process known as the Bergeron-Findeisen mechanism. In contrast, contact and immersion freezing are initiated when the IN approaches or becomes immersed in an aqueous solution or supercooled water droplet. Condensation freezing often takes place when three ice nucleating modes exist. Like AP activation, the IN ability to form IC varies depending on the type of particle and atmospheric sources and conditions. Regardless of source of IN, an increase in IN activation for temperature decrease of different sampling of air have been reported^10–12^.

Fundamental specific characteristics which are capable of adsorbing water molecules are the same features of ice nucleation activity. Experiments have shown that the geometrical structure of IN bonds at the substrate surface are as important as IC. For example, AgI with effective ice nucleability has similar structure to hexagonal IC. Similarly, good ice nucleability of kaolinite is also due to the pseudo-hexagonal arrangement of the hydroxyl groups at the surface of the lattice^10,12^. In the case of lattice mismatching, some unbounded molecules across the interface result in increasing interface free energy, consequently reducing the ability of the AP to serve as an IN.

Newly developed ideas suggest that certain locations (e.g., cracks, hydrophilic sites) of IN can act as an embryo for ice growth that is called active sites^10^. Some studies reported that the sites are less likely in smaller particles (less than 500 nm) to act as IN^11^; however, another study demonstrates that 200-nm diameter can constitute the majority of IN^12,13^. On the other hand, the existence of both hydrophilic and hydrophobic regions in the IN can facilitate surface diffusion of weakly adsorbed molecules near the active site^10^. Experimental studies also showed that the strength of the chemical bonds of the active sites affect their efficiency to exhibit good ice nucleability. Molecules with rotational symmetry at the substrate surface, whose active H-bonding groups are exposed to surface interactions, can build up effective active sites^12,14,15^. In addition, some chemical impurities presented onto the surface of IN can constitute or modify the effects of active sites. Adsorption of water molecules from the vapor showed, when AgI samples containing impurity ions such as K^+^ and No_3_^−^, have higher ice nucleation efficiency than pure AgI^10^. Some other studies revealed that IC also appear preferentially at cracks and in cavities where they could keep relative humidity below ice saturation due to the negative curvature effect^12,15–17^.

The organic molecular nature, rotational symmetry, hydrogen bonding groups matching to ice are of important features of active sites in the organic IN. There are some limited substances and biogenically driven organic aerosols in the atmosphere that can act as IN in Bergeron process^18,19^. Both primary and secondary organic particles have also been shown to nucleate ice despite their amorphous nature which seems to be in contrast to the concept of rotational symmetry and matching of hydrogen bonding groups^15^. On the other hand, studies on mineral materials showed quartz- and feldspar-containing of desert dust are preferential ice nucleating sites in the IN in mixed-phase cloud conditions at lower temperature (T < –15)^20,21^. interestingly, acidic treatment (with HNO_3_ and H_2_SO_4_) or coating of dust particles, containing kaolinite, illite, and feldspar, have suppressed IN activity in the deposition mode, which is in contrast to the effect of AgI impurity^10,12,22,23^. So, laboratory study of IN activity of idealized organic particles is a reliable approach to understand the complexities and contradictions in characteristics of the ambient organic and mineral IN activity.

New field study on black carbon (BC) particles as CCN showed the fact that the majority of them with a diameter greater than 180 nm have small BC cores with substantial coating through various atmospheric transport conditions. In addition, their mass size distribution typically peaks in the range of 120–170 nm, and for core mass equivalent diameter between 170 and 200 nm activation fraction increased more than 80% as the coating thickness increased from 0 to 60 nm^24^. Another study for different polarity and hydrophilicity of soot particles showed oxidation of thermal soot leads to an increase in the density of active water adsorption sites and the extent of surface polarity (intermediate polarity), and thus increases the hydrophilicity of the surface^15,25^. Uncoated soot particles of propane flame with low organic carbon content (5% organic carbon content) can build up more efficient IN than higher organic carbon content (30% and 70% organic carbon content), and up to 25% of these soot particles were activated in deposition mode for ice saturation ratio 1.22 at a temperature T = 226.6 K^25^.

There are some different mechanisms for water uptake of soot particles. First of all, hydrophilic soot, which contains relatively large amounts of water-soluble material, absorbed water through dissolution of these soluble materials. Secondly, organic composition AP in urban areas and diesel exhaust with different classes of polar compounds uptake water and, hence, form water droplet of insoluble soot cores. On the other hand, Non-polar organic compounds generally do not take up water and may also form films of water on the particles, thereby inhibiting adsorption or absorption of water molecules. Finally, water adsorption is observed on insoluble soot particles due to interaction with surface active sites^15,25^.

Recently graphene (G), a two-dimensional (2D) sp^**2**^ carbon network, has attracted a wide range of interest due to its fascinating electronic, mechanical and thermal properties after its empirical discovery in 2004. The single layer of G with a thickness of 0.35 nm consists of carbon atoms arranged in a hexagonal structure similar to IC structure. Thermal and chemical exfoliation method have been widely used in recent years and both of them are reliable and low-cost. The usual chemical and thermal reduction and oxidation may result in graphene oxide (GO) along with G, and they, in turn, make hydrophilic and hydrophobic islands inside nanoparticle^26–29^. This strange combination is desired in the HIN. Ice nucleation of such surfaces has been reported in zoology especially for Stenocara desert beetle that can harvest fog on the skin via such Nano physical process^30^. Other studies also show that different water-dispersible carbon nanomaterials are capable of nucleating ice, and this process is more efficient on tubes and curve structures than flat G species^31,32^. Here we use mixed GGO nanoparticles (GGON) in order to HIN in cloud chamber and probable cloud seeding.

In this study we used low temperature thermal shock (LTTS) reduction method on graphite oxide in order to obtain carbon-based nanoparticles. The resulting nanoparticles were identified using scanning electron microscope (SEM), Fourier transform infrared spectroscopy (FTIR) and Raman spectra of G sheets. Shape and size distribution of mixed GGON can be determined from image processing of the SEM results. The obtained size distribution is compatible with previous studies of soot and BC with precise differential mobility analyzer. Then GGON are introduced in cloud chamber that result in ice nucleation that is presented here. So, GGON properties from LTTS reductions method were investigated, whose combined hydrophobic and hydrophilic properties, chemical bounds and size distribution were considered in details.

This paper is organized as follows. The next section gives a brief overview of methods, materials and instrumentation that were used in this study. Cloud chamber features and material preparation are presented in next section. The third section presents SEM, FTIR, XRD and XRF and Raman spectra of the results. We try to confirm active sites onto the GGON and their 2D nature with the use of the results. Finally, the comparison between crystallization of commercial AgI, kaolinite and the GGON and their ice crystallization activity in the cloud chamber are presented.

## Materials and Methods

### Cloud Chamber

Cloud chambers are large temperature and humidity controlled vessels which are used to quantify amount and type of IC formation. A typical experiment involves the reduction of temperature and enhanced saturation ratio via expanding the water vapor in a chamber in order to form homogeneous cloud in specific conditions. In our experiments, cloud chamber consisted of usual chamber with volume of 220 L and dimensions of 906 × 555 × 840 mm. The chamber operated at atmospheric pressure and its temperatures can be set from −24 °C to warmer ranges. Specific amount of water vapour enters to the chamber via a hole at the bottom of it. Homogeneous temperature and humidity conditions throughout the volume can be controlled with electronic board. This condition was achieved by operating the mixing ventilator (small electric fan) in the chamber. After entering specific amount of water vapour to the chamber, it is completely filled by supercooled droplets in a few seconds. Then the particles were introduced in cloud chamber in order to study IC formation.

Ice crystals have a variety of shapes and sizes in different atmospheric conditions according to Magno-Lee classification^33^. But under condition of our experiment all type of plate-like IC were preferred. The IC in the chamber can be quantified onto the plates that were coated by Formvar (poly vinyl formal). After introducing IN in the chamber, IC gradually were formed during droplet disappearance, and finally just IC remained in the chamber. Ice crystals that were formed in different abovementioned modes were sampled with the replica technique^34^. The plate that was coated with a thin layer of different percentages of Formvar solution in chloroform (2%, 4% and 6%), were exposed to the falling crystals at the bottom of the chamber in specific time duration. These different percentages of Formvar were chosen due to different appearance of supercooled water droplets effects in different percentage of Formvar. In addition, we want to confirm that light transmit in center of IC for the GGON and observed IC asymmetry were not due to the coated conditions and compositions. Then IC concentrations in the chamber were obtained by counting them with an optical microscope, which was set outdoor of the chamber. The plates were placed inside a desiccator where IC and chloroform were evaporated. Finally, IC size distribution were obtained from image processing of the pictures. Even though this method is not very precise and updated, it can account for illustration of the GGON crystallization.

The humidity before IN nucleation, was measured by an electronic board (Arduino Uno microcontroller board) with 4 DHT22 sensors for controlling temperature gradient. Therefore, the sensors platform allows to get the current temperature and humidity of the chamber. Controlling temperature and humidity in the cloud chamber can be a difficult task in order to form homogeneous cloud in specific conditions. So, we suppose horizontal and vertical temperature gradient of around 1 K m^−1^ as homogeneous cloud. To ensure the possible influence of the walls on the nucleation process, the chamber’s wall was cleaned with acetone and alcohol every experiment and air filtration was conducted before cloud formation. The LTTS was done with the use of a small conical Nichrome wire (heater) with specific voltage for all materials, which was surrounded by small Pyrex glass tube in order to convey the resulting particles to the bottoms of the chamber. The temperature of the Nichrome heater ranged from room temperature up to 300 °C. Three types of available materials, which have the potential of HIN and cold cloud seeding, were injected in this way to the bottom of the chamber.

### Preparation of materials

The process of obtaining GGON by LTTS involves two main steps. First, we synthesized the graphite oxide by Hummer’s method based on the oxidize graphite powder. In this process, in a 250-cc flask 1 g of graphite was added to 25 cc of H_2_SO_4_. The flask was then placed in an ice bath for 15 min to cool it down to 0 °C. After that, 3 g of potassium permanganate (KMnO_4_) was added slowly to the flask. The obtained suspension was then stirred continuously for 2 h. The temperature in this step was kept at 35 °C. Subsequently, it was diluted by 200 cc of deionized water at the temperature 60 °C. H_2_O_2_ solution was then added to the flask over which the residual permanganate was reduced to soluble manganese ions. The obtained product was then isolated by filtration, washed copiously with deionized water, and dried at 60 °C for 24 h to obtain graphite oxide powder^35^.

**Figure 1:**
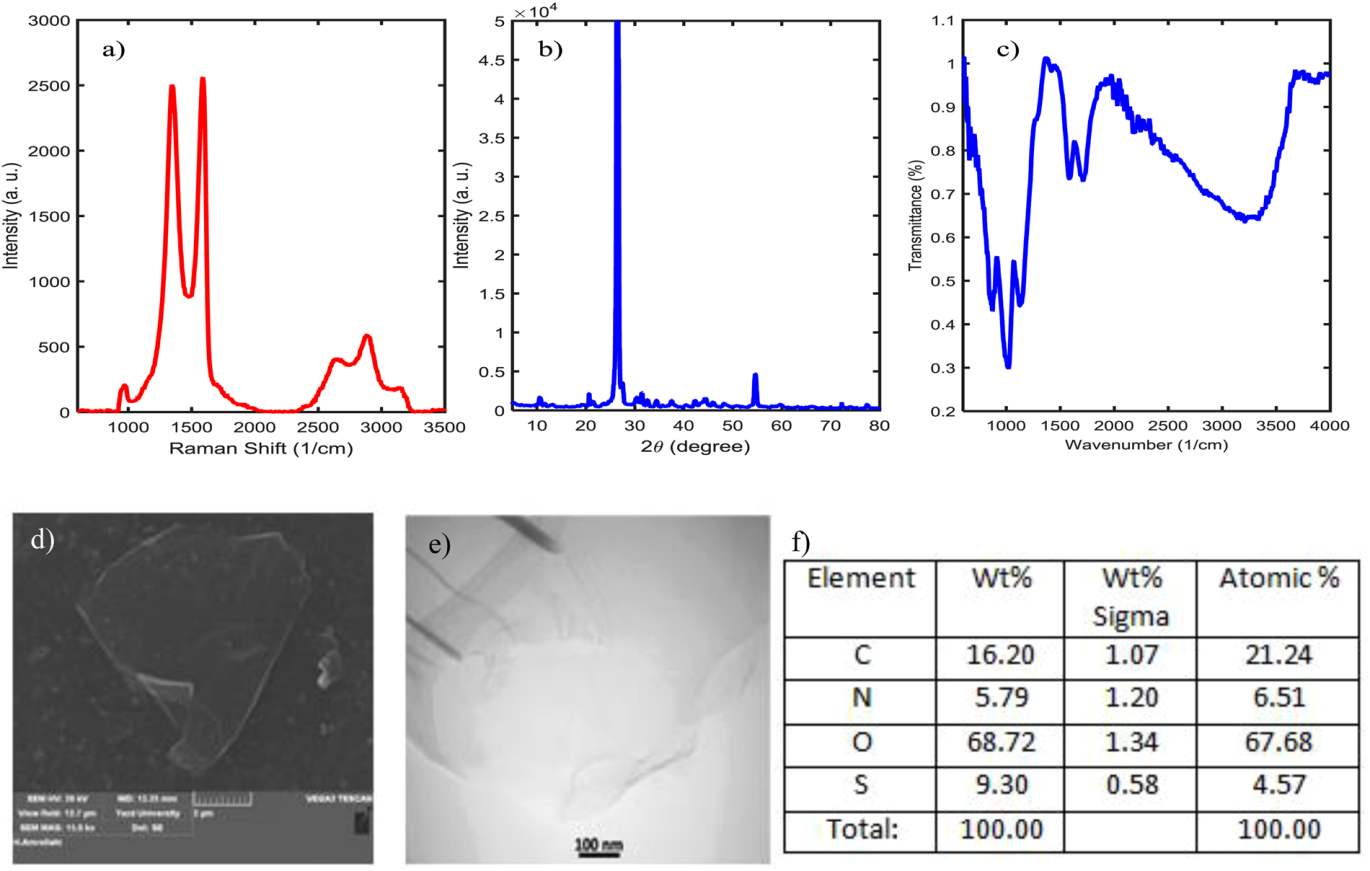
(**a**) Raman spectroscopy, (**b**) XRD (**c**) FTIR (for graphite oxide powder before thermal shock), (**d**) SEM, (**e**) TEM and of different type of GGO sheets, (**f**) Table of EDX for presence of some impurity in the GGO layer.

The Raman spectra of GGO layer exhibit two important peaks called D, G and 2D bands around 1331, 1590 and 2898 cm^−1^. The G band shows the presence of sp^2^ carbon-type structures within the sample, the D band is associated with the presence of defects in the hexagonal graphitic layers and 2D-band is attributed to the development of graphene structure (Fig. 1a). X-ray diffraction (XRD) has been used to study the morphology of GGO sheets and provided the peaks at 2θ = 10° (d001 = 0.83 nm), 2 θ = 20° (d002 = 0 0.48 nm), 2θ = 26° (d002 = 0 34 nm) and 2θ = 55° (d004 = 0 0.17 nm)^36^. The peaks at 2θ = 26°, 55° is characteristic of graphite and after chemical oxidation of it the inter planar distance increase and the other peaks appear (Fig. 1b). The distance between the GGO sheets are similar to the c-lattice constant of IC according to the XRD results.

The FTIR spectra show the presence of C-H, C-O, C-O-C, C-C and C = O at 900, 1020, 1123, 1583 and 1710 cm^−1^ respectively in Fig. 1c. The intensity of peaks is different for G layers and graphite oxide powder. Specially, the appearance of a spread peak in the region of 3000 to 3600 cm^−1^ is seen in FTIR of graphite oxide powder that is related to the tensile vibration of the O-H bond (Fig. 1c). The GGO nanoparticles show weaker peak in the similar vibration wavelength. The SEM and TEM imaging show the microstructure of synthesized GGO (Figs. 1d,e). The Table corresponding EDX analysis indicates the presence of some impurity in the GGO layer (Fig. 1f). The existence of No_3_^−^ is clear in this table.

**Figure 2:**
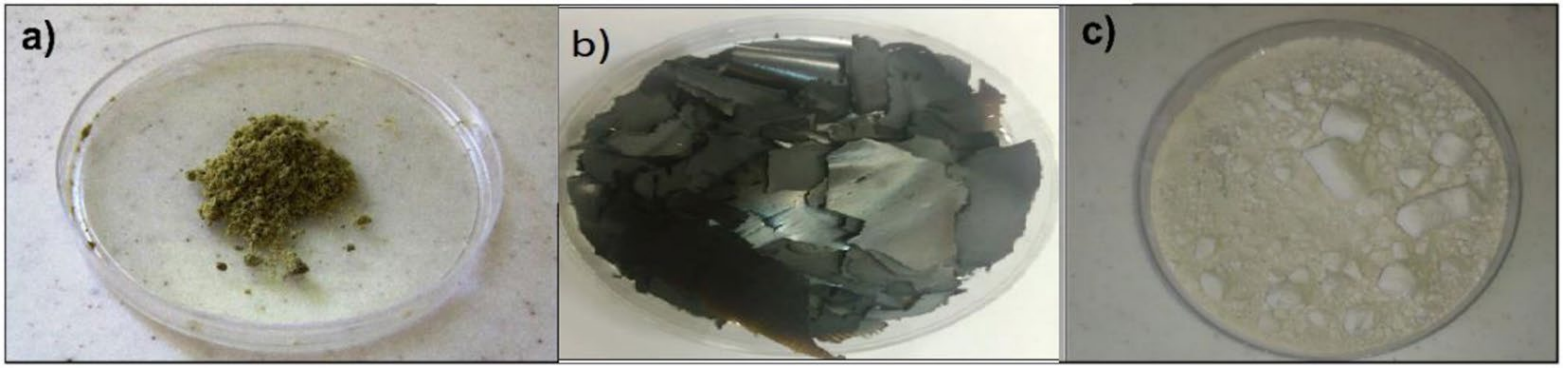
Silver iodide from PV-26 flares, (**b**) graphite oxide powder, (**c**) kaolinite powder.

Even though our main goal was considering IN activity of the GGON, we considered three types of available IN at the same condition in order to confirm and compare the obtained results. AgI, kaolinite and the GGON were used in our experiment in order to compare IN activity. We used the pyrotechnic mixture of the Russian flares PV-26, that is used in operational cloud seeding, which contains up to 4% AgI (Fig. 2a). The commercial AgI due to pyrotechnic materials can be easily burned in the Nichrome heater. We used commercial form of kaolin, which contained 47.35% SiO_2_ and 37% Al_2_O_3_. Some other impurities such as Na_2_O (1.11%), MgO (0.24%), CaO (0.65%), TiO_2_ (0.2%) and Fe_2_O_3_ (0.83%) also existed in the used kaolin (see Fig. 2c).

In the second step, the graphite oxide powder (Fig. 2b) was placed in the heater in order to perform fast LTTS, and the GGON in the cloud chamber were obtained. We used 0.01 g of abovementioned materials in the heater for introducing in the chamber in every experiment. One should note that thermal shock is a famous method to reduce GO, but it is very sensitive to the temperature gradient (°C /h). In this experiment, Nichorome got reddish in few seconds and the LTTS process has been occurred less than 10 s.

## Results and Discussions

**Figure 3:**
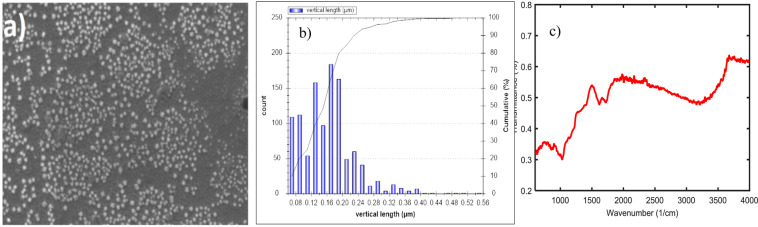
(**a**) SEM, (**b**) size distribution of the GGON derived from image processing and (**c**) FTIR of the GGON.

The resulting carbon-based nanoparticles are collected on prepared plate and image processing of SEM nanoparticles are performed with the use of the pre-specific size (Fig. 3a,b). Size distribution of the GGON and its characteristics with SEM is shown in Fig. 3. Size distribution of the GGON resulting from LTTS ranged from 80 nm to 400 nm with the maximum distribution around 160–180 nm (Fig. 3a,b). These GGO sheets in IN, where they make hydrophobic hexagonal island in hydrophilic the structure, can make active sites onto the IN and be activated in the cloud chamber. Formation of these active sites on the GGON are very important in HIN and cloud micro-physics, and mechanism of water adsorption on them should be clarified more. So, we try in the following section to describe such adsorption of water molecules from nanostructure view. In addition, Fig. 3c represents the epoxy and carboxyl group in the GGON by FTIR analysis.

According to results in Figs. 1 and 3 one can deduce both size distribution and structure of the GGON obtained from LTTS are of interest in HIN and IC formation. This distribution is compatible with previous aerosol counter measurements of BC (typically peaks in the range 120–170 nm^24^) to act as CCN and thermal soot size. The high ratio of O/C indicates the simultaneous presence of G and GO layer. Although G is hydrophobic, GO is hydrophilic and exist of these two properties side by side provides the benefit background for ice nucleation. In addition, research show that the fraction O/C ratio (here about 3) and low organic carbon have positive influence in active sites features and, hence, the ice nucleating activity.

Some impurity ions such as NO_3_^−^ are inserted into the graphene layers during the synthesis process, and they can enhance the probability of existence of active sites in GGON by increase the interlayer spacing and chemical adsorption. From this point of view, the GGON impurities can act similar to AgI impurities. The presence of functional groups like hydroxyl and carboxyl groups in the GGON results in adsorption of water molecules by hydrogen bonds. In fact, the water molecules have polar form near the surface of GGON as below:

$${{\rm{2H}}}_{2}{\rm{O}}\to {{\rm{OH}}}^{-}+{{\rm{H}}}_{3}{{\rm{O}}}^{+}$$

The positive ion bond with active group on the surface by withdrawing the electrons and negative ions can interact with positive part of another water molecule. Because of the random distribution of G and GO sheets with different willingness to attract the water, this mechanism may also be important in asymmetry of IC formation by the GGON. In addition, adsorbed water can penetrate through inner capillaries. Penetration of water down can increase the adsorption strength and distance between layer of GGO. As reported previously some researches demonstrated cracks, cavity and edges of substrate can result in active sites formation in the IN, so the capillaries of GGO can act in the same manner. The distance between the GGO sheets are similar to the c-lattice constant of IC according to the XRD results. Also the hexagonal lattice and fractal properties of GGO cause to crystal matching between them and ice crystal that has main effect on the nucleation ability.

**Figure 4:**
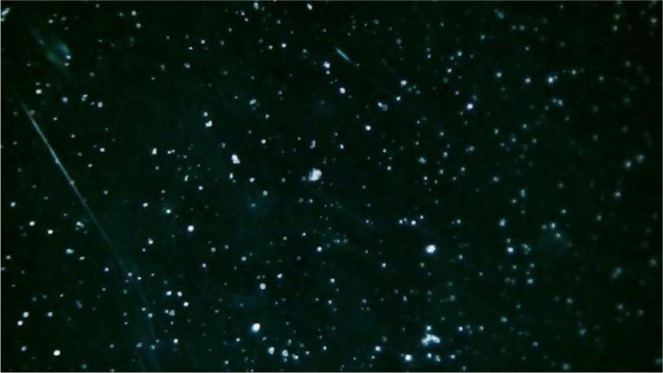
IC with the use of GGON in the cloud chamber.

**Figure 5:**
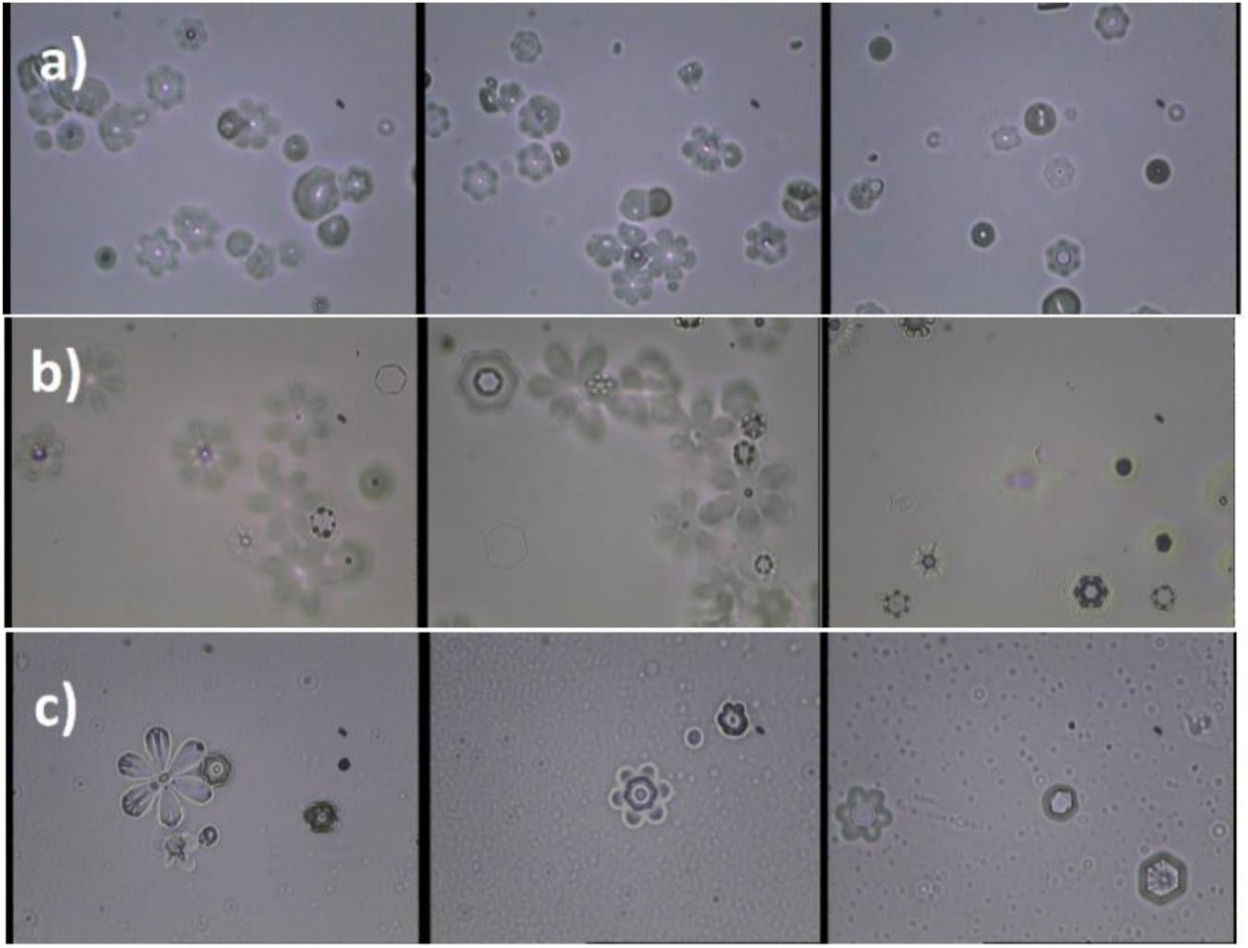
Replica of IC formed on slides covered with formvarpoly vinyl formal and 2% chloroform (first column), 4% chloroform (middle column) and 6% chloroform (last column), (**a**) GGON (**b**) AgI (**c**) Kaolinite.

According to these nanostructure description, GGO sheets in the GGON, where they make hydrophobic hexagonal island in the hydrophilic structure, can make active sites in the IN and be activated in the cloud chamber (Fig. 4). The formation of IC by the GGON in the cloud chamber is presented in the Fig. 4. Here, three specific cloud conditions in T = −10 °C, −15 °C and −20 °C have been selected in the cloud chamber during importing the GGON in order to study temperature effects on IC formation. The effects of three types of the IN (the GGON in the first row, AgI and kaolinite in second and third row) are shown for comparison in Fig. 5. Figure 5a shows the IC of GGON, in which their sizes and shapes are different from other two materials (compare Fig. 5a with 5b and 5c). Their sizes are smaller than IC of AgI and kaolinite. The center of IC of GGON transmit the underlay visible light in all percentages of Formvar solution, but this effect was not seen in IC of other materials. This is due to famous optical features of GGO, in which GGO transmit more than 95% of visible light^37^.

There are also some types of asymmetry and deformation in the IC formation of GGON. The skewness in some crystals structure can be seen in Fig. 5a. some probable reason for this effect was declared from nanostructure point of view. A type of asymmetry in IC had been reported previously for spruce budworm antifreeze protein at atmospheric pressure. They interpreted this asymmetry in terms of growth-melt-growth sequences, in which both growth and melt shapes of ice faceted hexagonal morphology, are rotated 30° relative to each other^38^. But this asymmetry or deformation the IC of GGON is not regular and ordered. Here a type of directional asymmetry that tend to be circular at the edge of IC was seen. Some circular large droplets also existed in Fig. 5a that is similar to droplet formation that their centers do not transmit light (graphite particles). So different mechanisms may be contributed in IC and activation of GGON. From one hand, the LTTS can result in not only GGON, but also some graphite particles are generated. On the other hand, some active sites of GGON may contain hygroscopic graphite sites. Both of these probable mechanisms can result in asymmetry in the IC. In the former mechanism, IN growth in active sites of GGON and some circular shapes are due to water absorption of hygroscopic graphite. Then the coagulation them can justify the observed IC. The droplets are also darker than the IC due to rapid freezing of supercooled droplet, when they collide to graphite particles. Because when supercooled droplets freeze immediately on the IN, producing a coating of opaque rime ice containing many air bubbles. The later mechanism can result in asymmetry and deformation of an IN, in which it tends to grow like a crystal in one aspect and activate like a droplet in another aspect. This interpretation is also compatible with XRD results, in which the peaks at 2θ = 26°, 55° are characteristic of graphite. The effects of supercooled droplets on plates that are more apparent in more percentages of chloroform (4% and 6%) cannot be observed for GGON. That means most of the cloud’s droplets in the chamber can change into IC or larger droplets.

**Figure 6:**
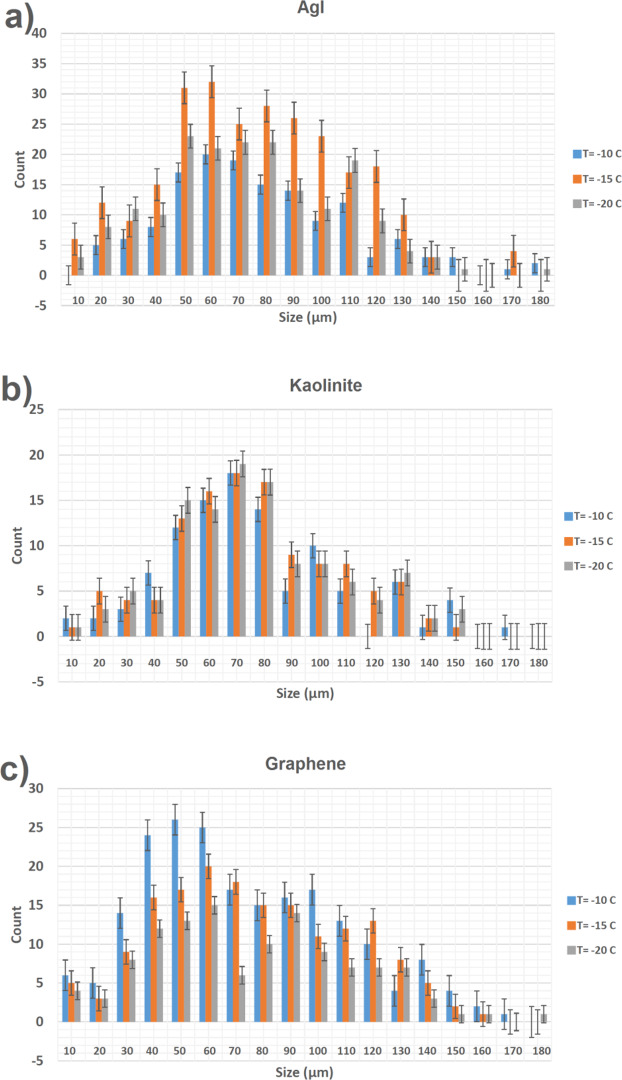
Size distribution of IC with (**a**) Silver iodide. (**b**) Kaolinite, (**c**) G and GO nanoparticles.

The size distribution of IC in the chamber are shown in Fig. 6 in three different temperatures, T = −20 °C, −15 °C and −10 °C. Results show that different behave different at these temperatures. HIN is comparable with AgI. Circular diameter and size distribution of crystals that exist in silver iodide has been measured and result can be seen in Fig. 6a. Circular diameters of these crystals are in range between 10–180 µm. The maximum range of diagram is 50 µm to 100 µm. Circular diameter and size distribution of crystals that exist in Kaolinite has been measured and result can be seen in Fig. 6b. Circular diameters of these crystals are in range between 10-180 µm. The maximum range of diagram is 50 µm to 80 µm. Circular diameter and size distribution of crystals that exist in the GGON has been measured and result can be seen in Fig. 6c. Circular diameters of these crystals are in range between 10-180 µm. The maximum range of IC occur at −10 °C and IC concentration increased as temperature increased. This effect is probably due to increase in concentration of IC with hygroscopic graphite particles water uptake that formed during contact mode in warmer ranges.

## Conclusion

The ice nucleation usually requires active sites in the aerosols in order to act as an IN. Both chemical and mechanical features of substrate surface are important in building up active sites in the IN. Consequently, impure ions and cracks, cavities and capillaries result in formation of active sites. In this study, HIN and IC formation on probable active sites of the GGON, obtained from graphite oxide by LTTS, were considered. Size distribution of the GGON ranged from 80 nm to 400 nm with the maximum distribution around 160–180 nm, which is compatible with previous aerosol counter measurements of BC (typically peaks in the range of 120–170 nm^24^) and thermal soot size (core size distribution pre-expansion of 150–250 nm^15,25^).

The FTIR spectra show the presence of C-H, C-O, C-O-C, C-C for G layers and graphite oxide powder, in that tensile vibration of the O-H bond of graphite oxide in the region of 3000 to 3600 cm^−1^ illustrate spread peak. The Raman spectra of GGO sheets exhibit two important peaks called D, G and 2D bands, where G band shows the presence of sp^2^ carbon-type structures within the sample, the D band is associated with the presence of defects in the hexagonal graphitic layers and 2D-band is attributed to the development of graphene structure. The GGON contained GGO sheets, where desired active sites of hydrophobic hexagonal island are placed in the hydrophilic structure. Although G is hydrophobic, GO is hydrophilic and the existence of these two properties side by side provides the benefit background for ice nucleation. The distance between sheets in the GGO sheets are similar to the c-lattice constant of IC according to the XRD results. Also, the hexagonal lattice and fractal properties of GGO cause crystal matching between them and ice crystal that has main effect on the nucleation ability. After introducing these carbon-based particles in the cloud chamber by LTTS, IC were formed. According to EDX and results, some impurity ions such as No_3_^−^ are inserted into the graphene layers during the synthesis process, and similar to AgI, they can enhance the probability of existence of active sites in the GGON by increasing the interlayer spacing and chemical adsorption. In addition, adsorbed water can penetrate through inner capillaries. Penetration of water down can increase the adsorption strength and distance between layer of GGO.

The IC of the GGON are smaller than IC of AgI and kaolinite. The center of IC of the GGON transmit the underlay visible light in all percentages of Formvar solution. This is due to famous optical features of the GGO sheet, in which the GGO transmit more than 95% of visible light^35^. There are also some types of asymmetry and deformation in IC formation of the GGON. A type of asymmetry that tend to be circular at the edge was seen. Some circular large droplets also existed that their centers do not transmit the underlay light due to different structure of the graphite particles. Two possible mechanisms were proposed for the seen asymmetry or deformation in IC formation of the GGON. First, LTTS can result in not only the GGON, but also some graphite particles. Then the IN grow in active sites of the GGON and some droplets are formed due to water absorption of hygroscopic graphite. Then the coagulation and collision of them can justify the observed IC. Second, some active sites of the GGON may contain hygroscopic graphite sites. This mechanism can result in asymmetry and deformation in an IN, in which it tends to grow like a crystal from one aspect and activate like a droplet in the another aspect. This interpretation is also compatible with XRD results, in which the peaks at 2θ = 26°, 55° are characteristic of graphite.

In addition to fascinating electronic, mechanical and thermal properties of GGO sheets, their HIC of 2D nanoparticles should be studied more. Some of their interesting properties and possible mechanisms for IC formation were presented. Because of lack of laboratory instruments for optical aerosol size distribution, CCN counter, CPC and SP2, we cannot determine precise activation fraction of the GGON in different SS rations. Complementary studies can be done with the use of SP2. So, due to importance of the carbon type particles in many phenomena, such as forest fires and volcanic ash and their effects on climate change, we propose to continue such 2D carbon based nanoparticles in big and famous cloud chambers with enough updated instruments. In addition, probable formation of 2D carbon particles during forest fires and volcanic eruptions were also proposed.

## Supplementary information

Supporting information.
