# Supplementary material for: Heterogeneous Ice Nucleation by Graphene Nanoparticles
Source: Sci Rep. 2020 Jun 16;10:9723. doi: 10.1038/s41598-020-66714-2 (PMC7298023; doi:10.1038/s41598-020-66714-2)
Supplement: Supplementary file 1 — Supporting information. [file 41598_2020_66714_MOESM1_ESM.docx]

Supporting Information for “Heterogeneous Ice Nucleation by Graphene Nanoparticles”

Mohammad Joghataei*^1^, Fatemeh Ostovari^1^, Samira Atabakhsh^1^& Nafiseh Tobeiha^1^

1-Department of Physics, Yazd University, Yazd, Iran

* Corresponding author: Mohammad Joghataei ([mjoghataei@yazd.ac.ir](mailto:mjoghataei@yazd.ac.ir))

**Contents**

1. introduction

2. supplementary Figures for Fig.3

3. supplementary descriptions for Fig.5

4. supplementary Figure for Fig.6

5. Supplementary for Shape influence

1 Introduction

To describe more about accuracy of image processing, we present other aspect of our process here. For example, for Fig.3 of results, we can choose horizontal and vertical length separately and present both of them. But for short we just present vertical length in the manuscript. As you can see in the below figures, there is no significant difference between size distribution of horizontal and vertical length. So, the obtained GGON are approximately circular. In addition, we try to justify the hydrophilicity of the GGON via FTIR and other analysis. So, the GGON contained GGO sheets, where desired active sites of hydrophobic hexagonal island are placed in the hydrophilic structure. Although G is hydrophobic, GO is hydrophilic and the existence of these two properties side by side provides the benefit background for ice nucleation.

2 supplementary Figures for Fig.3

| 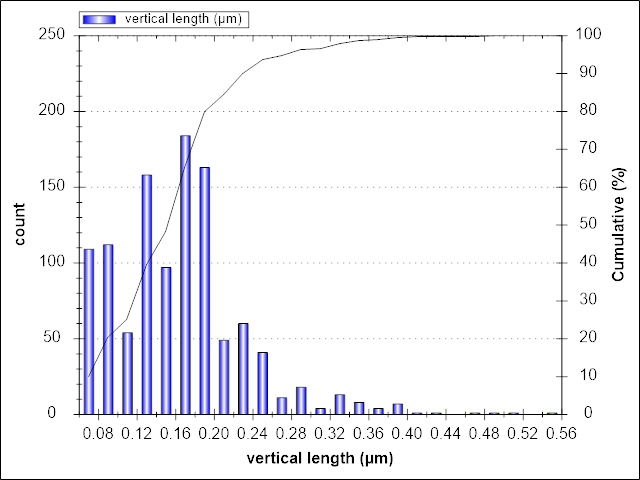  b)  b) | 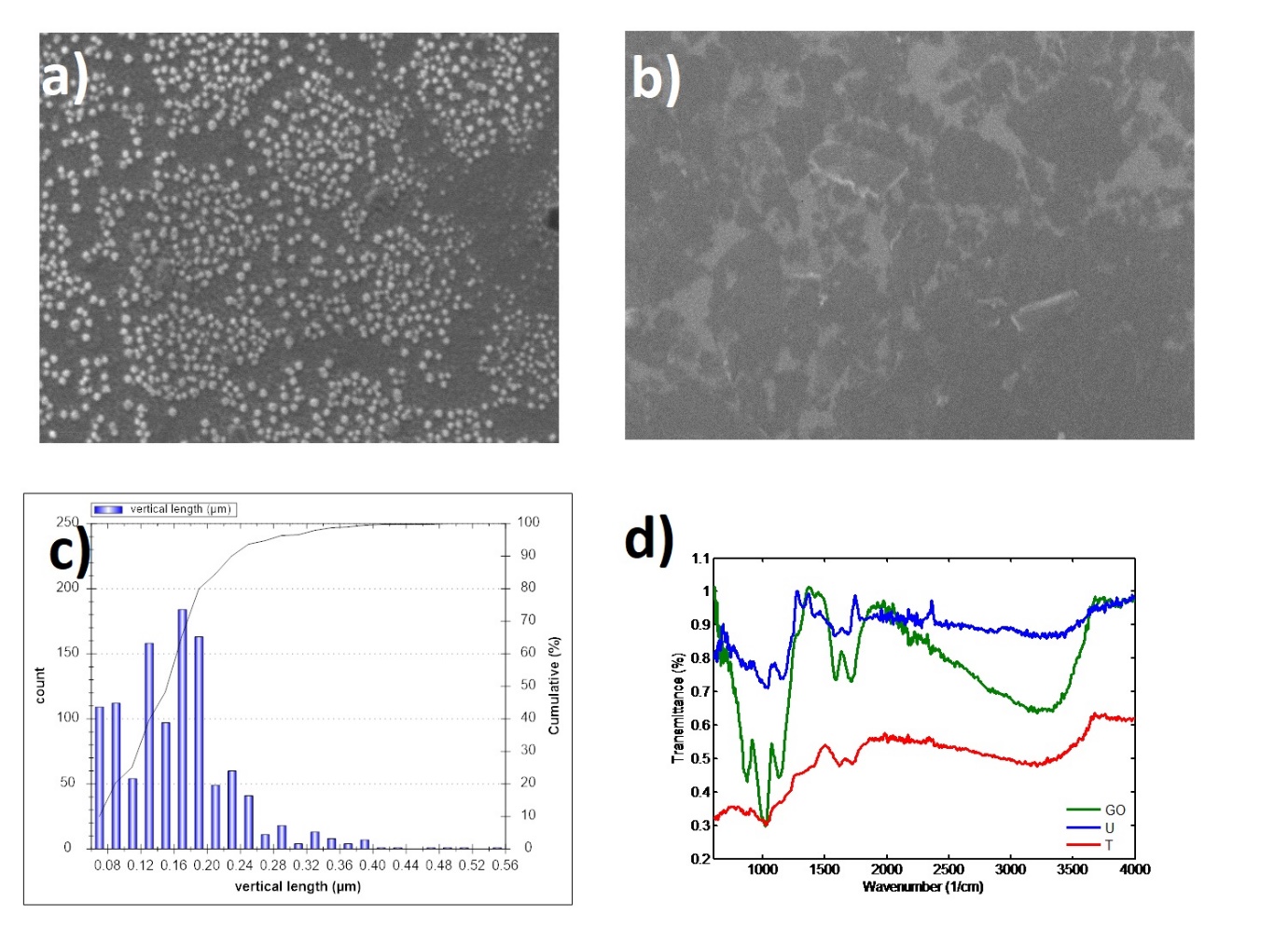 |
| --- | --- |

Figure 3 of the manuscript a) main picture b) image processing of (a).

| 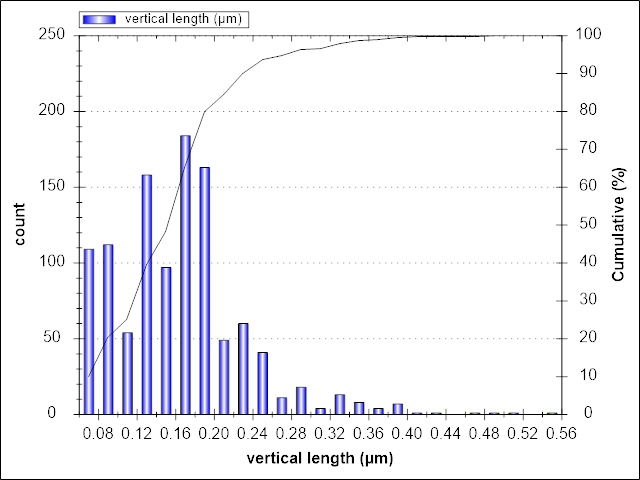  b) | 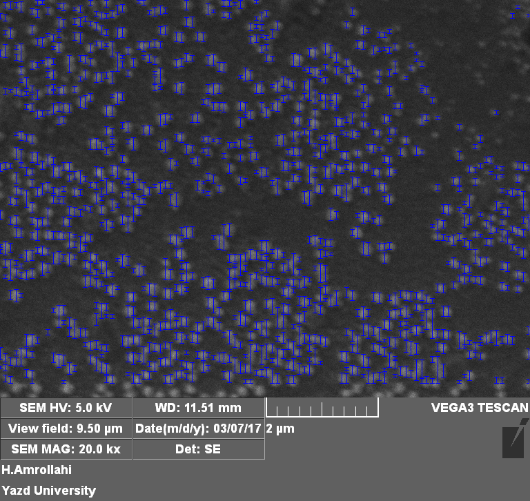  a) |
| --- | --- |

Supplementary for Fig.3 Vertical length of the GGON a) main picture b) image processing of (a).

| 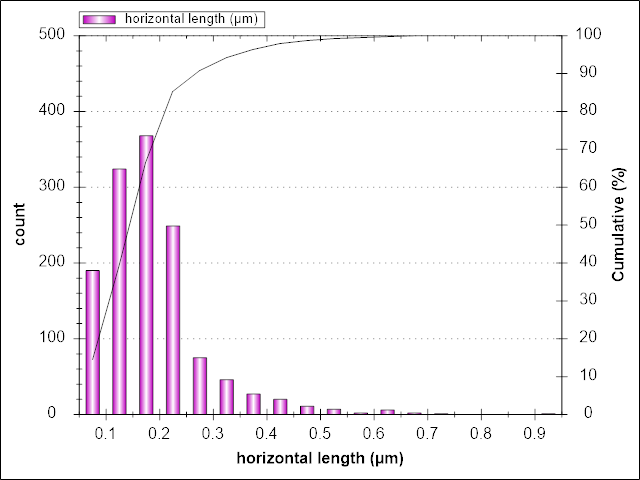 | 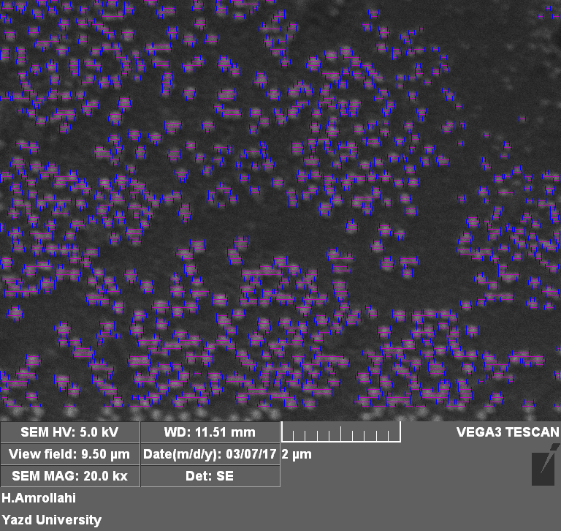 a)  b) |
| --- | --- |

Supplementary for Fig.3 Horizontal length of the GGON a) main picture b) image processing of (a).

The accuracy of image processing depends directly on the accuracy of scale of the SEM results. For more details about the results of SEM with scale bar, view field, beam energy and date. In some figure some grains are sized with the SEM software. For image processing, we define the scale bare as pre-specific length. For short, these scale bars are cropped for more ordered and tidy figures. Here, we present some other SEM results (not presented in the manuscript):

| 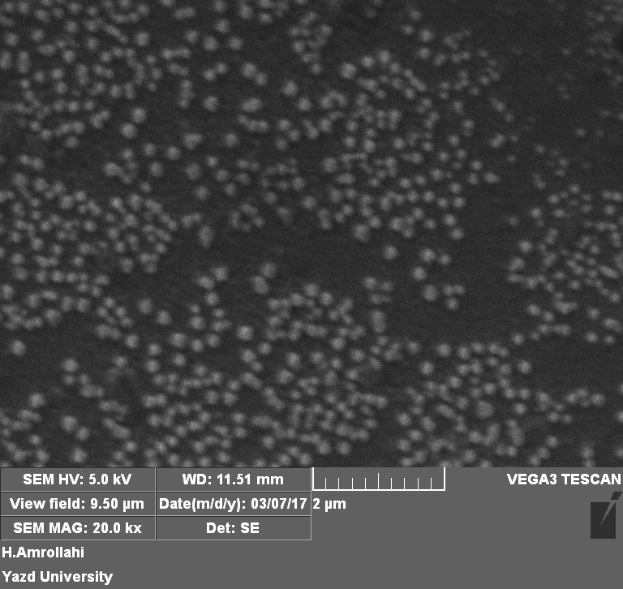 | 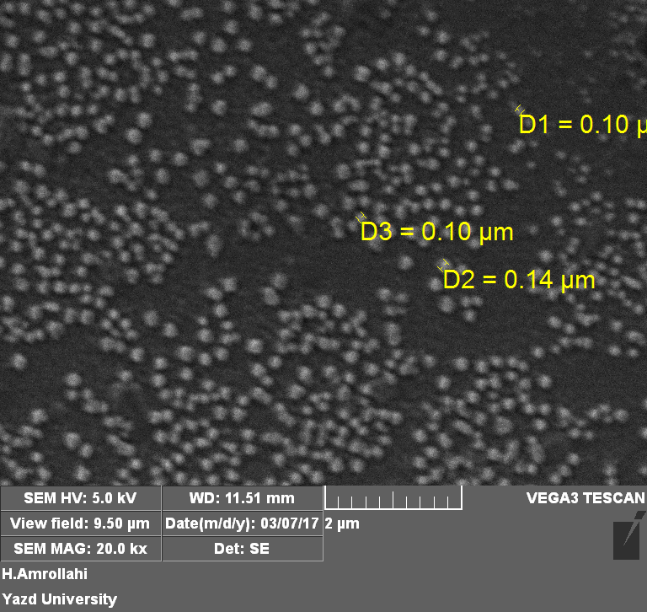 |
| --- | --- |
|  |  |
| 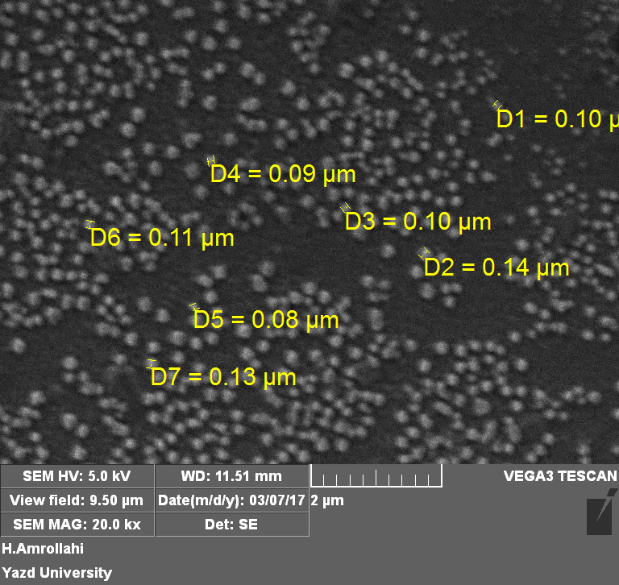 | 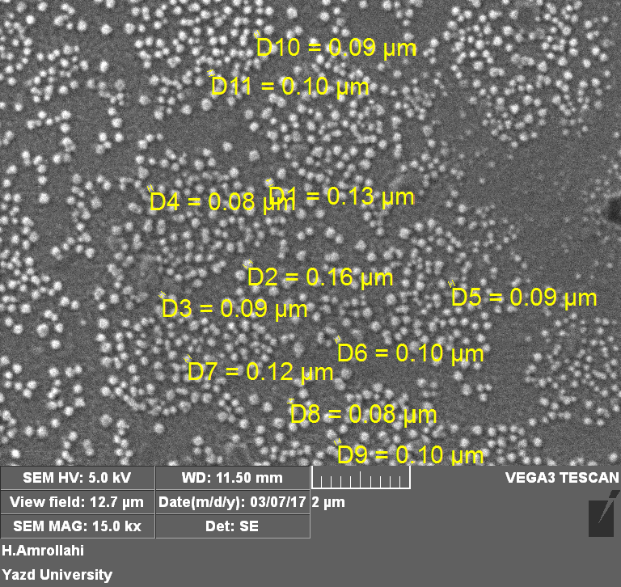 |

Supplementary Fig3. More SEM results with determining some of the GGON size with SEM software.

3 Supplementary descriptions for Figure 5

These different percentages of Formvar were chosen due to different appearance of supercooled water droplets effects in different percentage of Formvar. The effects of supercooled droplets on plates that are more apparent in more percentages of chloroform (4% and 6%) cannot be observed for GGON. In addition, we want to confirm that light transmit in center of IC for the GGON and observed IC asymmetry were not due to the coated conditions and compositions. Then IC concentrations in the chamber were obtained by counting them with an optical microscope, which was set outdoor of the chamber. The plates were placed inside a desiccator where IC and chloroform were evaporated. Finally, IC size distribution were obtained from image processing of the pictures. Even though this method is not very precise and updated, it can account for illustration of the GGON crystallization.

4 supplementary Figure for Fig.6

For determination of scale bar for IC we take some SEM from plates. One of them are presented below:


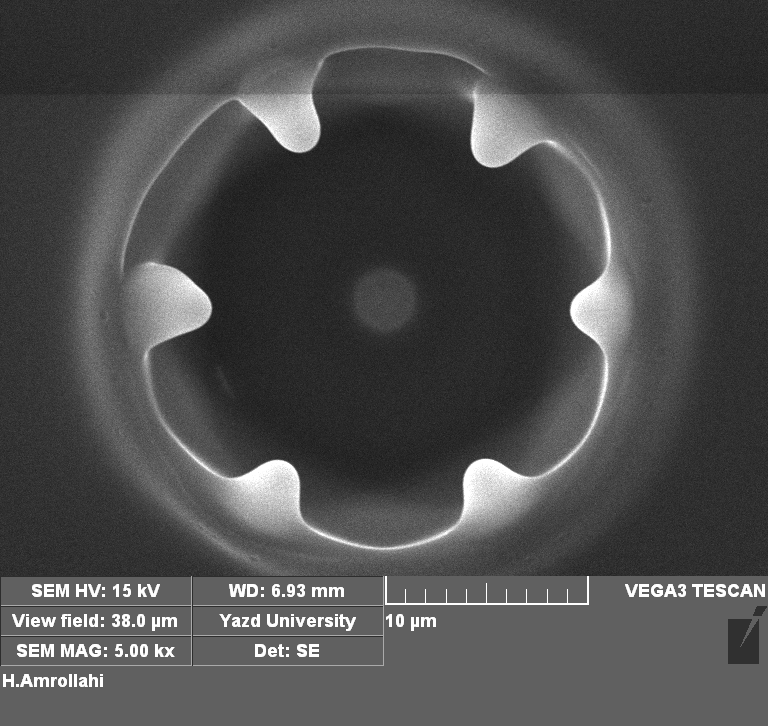


Supplementary of Fig. 6 SEM of IC for determination of scale bar for IC size distribution’s error bar. The halo region around IC related to error bar.

Supplementary for Shape influence

Homogeneous nucleation is much rarer than heterogeneous nucleation. However, homogeneous nucleation is simpler and easier to understand than heterogeneous nucleation, so the easiest way to understand heterogeneous nucleation is to start with homogeneous nucleation. So we will outline the classical nucleation theory (CNT) calculation for the homogeneous nucleation barrier Δ G ∗ {\displaystyle \Delta G^{*}} ∆G^*^.

To understand if nucleation is fast or slow, ∆G(r) Δ G ( r ) {\displaystyle \Delta G(r)} needs to be calculated. The classical theory assumes that even for a microscopic nucleus of the new phase, we can write the free energy of a droplet ∆G Δ G {\displaystyle \Delta G} as the sum of a bulk term that is proportional to the volume of the nucleus, and a surface term, that is proportional to its surface area

$$\Delta G=\frac{4}{3}\pi r^{3}\Delta g+4\pi r^{2}\sigma$$

The first term is the volume term, and as we are assuming that the nucleus is spherical, this is the volume of a sphere of radius r {\displaystyle r} r.$\Delta g$ Δ g {\displaystyle \Delta g} is the difference in free energy per unit volume between the thermodynamic phase nucleation is occurring in, and the phase that is nucleating. For example, if water is nucleating in supersaturated air, then Δ g {\displaystyle \Delta g} $\Delta g$ is the free energy per unit volume of the supersaturated air minus that of water at the same pressure. As nucleation only occurs when the air is supersaturated, Δ g {\displaystyle \Delta g} $\Delta g$ is always negative. The second term comes from the interface at surface of the nucleus, which is why it is proportional to the surface area of a sphere. σ {\displaystyle \sigma } σ is the [surface tension](https://en.wikipedia.org/wiki/Surface_tension) of the interface between the nucleus and its surroundings, which is always positive.

For small r {\displaystyle r} r the second surface term dominates and Δ G ( r ) > 0 {\displaystyle \Delta G(r)>0} $\Delta G\left( r \right)>0$. The free energy is the sum of an r 2 {\displaystyle r^{2}} r^2^ and r 3 {\displaystyle r^{3}} r^3^ terms. Now the r 3 {\displaystyle r^{3}} r^3^ terms varies more rapidly with r {\displaystyle r} r than the r 2 {\displaystyle r^{2}} r^2^ term, so as small r {\displaystyle r} r the r 2 {\displaystyle r^{2}} r^2^ term dominates and the free energy is positive while for large r {\displaystyle r} r, the r 3 {\displaystyle r^{3}} r^3^ term dominates and the free energy is negative. This shown in the figure to the right. Thus at some intermediate value of r {\displaystyle r} r, the free energy goes through a maximum, and so the probability of formation of a nucleus goes through a minimum. There is a least-probable nucleus occurs, i.e., the one with the highest value of $\Delta G$ Δ G {\displaystyle \Delta G} where

$$\frac{dG}{dr}=0$$

This is called the critical nucleus and occurs at a critical nucleus radius

$$r^{*}=-\frac{2\sigma}{\Delta g}$$

Addition of new molecules to nuclei larger than this [critical radius](https://en.wikipedia.org/wiki/Critical_radius) decreases the free energy, so these nuclei are more probable. The rate at which nucleation occurs is then limited by, i.e., determined by the probability, of forming the critical nucleus. This is just the exponential of minus the free energy of the critical nucleus Δ G ∗ {\displaystyle \Delta G^{*}}${\Delta G}^{*}$, which is

$${\Delta G}^{*}=\frac{16\pi\sigma^{3}}{3{(\Delta G)}^{2}}$$

This is the free energy barrier needed in the [CNT](https://en.wikipedia.org/wiki/Classical_nucleation_theory#Outline_of_classical_nucleation_theory) expression for R {\displaystyle R} R above.

Heterogeneous nucleation, nucleation with the nucleus at a surface, is much more common than homogeneous nucleation. Heterogeneous nucleation is typically understood to be much faster than homogeneous nucleation using classical nucleation theory. This predicts that the nucleation slows exponentially with the height of a [free energy](https://en.wikipedia.org/wiki/Gibbs_free_energy) [barrier](https://en.wikipedia.org/wiki/Activation_energy#Relationship_with_Gibbs_energy) ΔG*. This barrier comes from the free energy penalty of forming the surface of the growing nucleus. For homogeneous nucleation the nucleus is approximated by a sphere but as we can see in the schematic of macroscopic droplets to the right, droplets on surfaces are not complete spheres and so the area of the interface between the droplet and the surrounding fluid is less than a sphere's$4\pi r^{2}$4 π r 2 {\displaystyle 4\pi r^{2}} . This reduction in surface area of the nucleus reduces the height of the barrier to nucleation and so speeds nucleation up exponentially.

Any way due to limitation of words in the journal we just discussed about active sites and their influence on HIN. In addition, the GGON are approximately spherical that is in consistent with CNT too. for additional information of CNT and some point about “spherical ice germs” that are preferential in CNT, see page 198 of reference 10.
